# Multi-institutional survey of antiemetic therapy in lung cancer patients treated with carboplatin in Hokushin region

**DOI:** 10.1186/s12890-023-02524-2

**Published:** 2023-06-26

**Authors:** Takayuki Ide, Yoshikazu Nishino, Tomoya Takiguchi, Shintaro Kanda, Kengo Otsuki, Ryuji Hayashi, Kazuo Yasumoto, Yasuo Hirono, Tomoe Makino, Seiji Yano, Tomonobu Koizumi

**Affiliations:** 1 Department of Pharmacology, Shinshu University School of Medicine, Matsumoto, Japan; 2 Department of Epidemiology and Public Health, Kanazawa Medical University, Uchinada, Ishikawa, Japan; 3 Department of Hematology and Medical Oncology, Shinshu University School of Medicine, 3-1-1 Asahi, 390-8621 Matsumoto, Nagano, Japan; 4 Clinical Oncology, Toyama University Hospital, Toyama, Japan; 5 Department of Medical Oncology, Kanazawa Medical University, Uchinada, Ishikawa, Japan; 6 Cancer Care Promotion Center, University of Fukui Hospital, Fukui, Japan; 7 Division of Adult Nursing Practice, Ishikawa Prefectural Nursing University, Kahoku, Japan; 8 Division of Medical Oncology, Cancer Research Institute, Kanazawa University, Kanazawa, Japan

**Keywords:** Hospital-based cancer registry, Chemotherapy-induced nausea and vomiting, Antiemetic guideline, 5-hydroxytryptamine-3 receptor antagonist, Neurokinin-1 receptor antagonist

## Abstract

**Objective:**

Appropriate monitoring and management of chemotherapy-induced nausea and vomiting (CINV) with prophylactic antiemetics is important for cancer patients. This study was performed to validate the clinical practice of antiemetic use with carboplatin-based chemotherapy in lung cancer patients in the Hokushin region (Toyama, Ishikawa, Fukui, and Nagano prefectures), Japan.

**Methods:**

We surveyed retrospective data of newly diagnosed and registered lung cancer patients initially treated with carboplatin-based chemotherapy in 21 principal hospitals in the Hokushin region linked with health insurance claims data between 2016 and 2017.

**Results:**

A total of 1082 lung cancer patients (861 [79.6%] men, 221 [20.4%] women; median age 69.4 years [range, 33–89 years]). All patients received antiemetic therapy, with 613 (56.7%) and 469 patients (43.3%) receiving 5-hydroxytryptamine-3 receptor antagonist/dexamethasone double regimen and 5-hydroxytryptamine-3 receptor antagonist/dexamethasone/neurokinin-1 receptor antagonist triple regimen, respectively. However, the rates of double regimen and use of palonosetron were higher in Toyama and Fukui prefectures. Thirty-nine patients (3.6%) changed from double to triple regimen, while 41 patients (3.8%) changed from triple to double regimen after the second cycle, but six of these returned to triple antiemetics in subsequent cycles.

**Conclusion:**

Adherence to antiemetic guidelines in clinical practice was high in Hokushin region. However, rates of double and triple antiemetic regimens differed between the four prefectures. Simultaneous analysis of nationwide registry and insurance data was valuable for evaluating and comparing the differences in the status of antiemesis and management.

**Supplementary Information:**

The online version contains supplementary material available at 10.1186/s12890-023-02524-2.

## Introduction

Chemotherapy-induced nausea and vomiting (CINV) is a serious adverse event associated with chemotherapy. CINV often reduces quality of life, adherence to treatment, treatment efficacy, and curability in patients with malignancies. Several international clinical guidelines for antiemetic treatments recommend prescriptions based on the emetic risk of the chemotherapeutic agent used [1–4]. Prophylactic triple antiemetic therapy consisting of 5-hydroxytryptamine-3 receptor antagonist, dexamethasone, and neurokinin-1 receptor antagonist was recommended for highly emetogenic chemotherapy (HEC), while double antiemetic therapy with 5-hydroxytryptamine-3 receptor antagonist and dexamethasone was recommended for moderately emetogenic chemotherapy (MEC).

Carboplatin has been classified as MEC, but was reported to have the highest risk of CINV among patients receiving MEC [1–4]. Therefore, the guidelines and clinical studies suggest administration of antiemetics according to the recommendations for the HEC classification [1–9]. The 2015 Japanese Society of Clinical Oncology (JSCO) guidelines for CINV also recommended addition of neurokinin-1 receptor antagonist (aprepitant) to carboplatin-based chemotherapy, although selection of the addition of neurokinin-1 receptor antagonist was optional and left to the discretion of the attending physician [1].

A combination of health service utilization and hospital-based cancer registry (HBCR) data in Japan revealed a relatively high rate of compliance with CINV guidelines in clinical practice in Japan [10], but 8% of patients receiving intravenous MEC were treated without any antiemetics. Real-world prescribing data in Europe indicated low adherence to antiemetic guidelines and that 19% of patients were treated without prophylactic antiemetics in carboplatin-based chemotherapy [11]. Therefore, to further assess antiemetic prophylaxis, information about use of antiemetics in clinical practice, especially in carboplatin-based chemotherapy, should be evaluated. Furthermore, little information was available regarding the frequency and/or status of adding neurokinin-1 receptor antagonist to carboplatin-based chemotherapy in clinical practice.

The present study was focused on evaluating the preventive antiemetic status in lung cancer patients treated with carboplatin-based chemotherapy in Hokushin region, Japan. We surveyed retrospective data of patients with lung cancer using the Hokushin Ganpro Database and health care utilization data, and evaluated clinical practice patterns for prevention of carboplatin-related CINV in patients with lung cancer in Hokushin region, Japan.

## Materials and methods

### Hokushin ganpro database and health care utilization data

The Hokushin region of Japan is composed of four prefectures: Fukui, Ishikawa, Toyama, and Nagano prefectures (Supplemental Fig. 1). “Hokushin Ganpro” is an educational program implemented by the Ministry of Education, Culture, Sports, Science and Technology of Japan (https://gan-pro.net/) to enable improved cancer treatment by training highly skilled health care professionals via cooperation among the universities in the Hokushin region (Kanazawa University, Kanazawa Medical University, Shinshu University, The University of Toyama, The University of Fukui, and Ishikawa Prefectural Nursing University). The Hokushin Ganpro Database is a regional cancer database created as one of the projects of the Hokushin Ganpro and built from the HBCR of designated cancer care hospitals. Based on this database, we previously reported the several studies, including surveys of rare tumors [12], disabled patients [13], and pediatric and adolescent and young adult populations [14] in the Hokushin region. We recently launched an observational study of a regional cancer database from January 1, 2016, through December 31, 2017, as dataset 2 of the Hokushin Ganpro Database. Dataset 2 included health care utilization data, so-called Diagnosis Procedure Combination (DPC) survey data, in the Hokushin region. Collection of DPC data was performed as part of a governmental survey to assess the effects of the introduction of the DPC combination-based payment system. The survey data included information equivalent to fee-for-service insurance claims covering all billable health services (e.g., diagnostic tests, imaging workup, procedures, treatments, and prescribed drugs) for both inpatients and outpatients. These data were linked to the HBCR data of each patient in the participating hospitals. The Hokushin region has 28 designated cancer care hospitals in which approximately 35, 000 people are diagnosed with cancer and registered every year. Among them, 21 hospitals participated in Hokushin Ganpro dataset 2 (Supplemental Table 1).

**Fig. 1:**
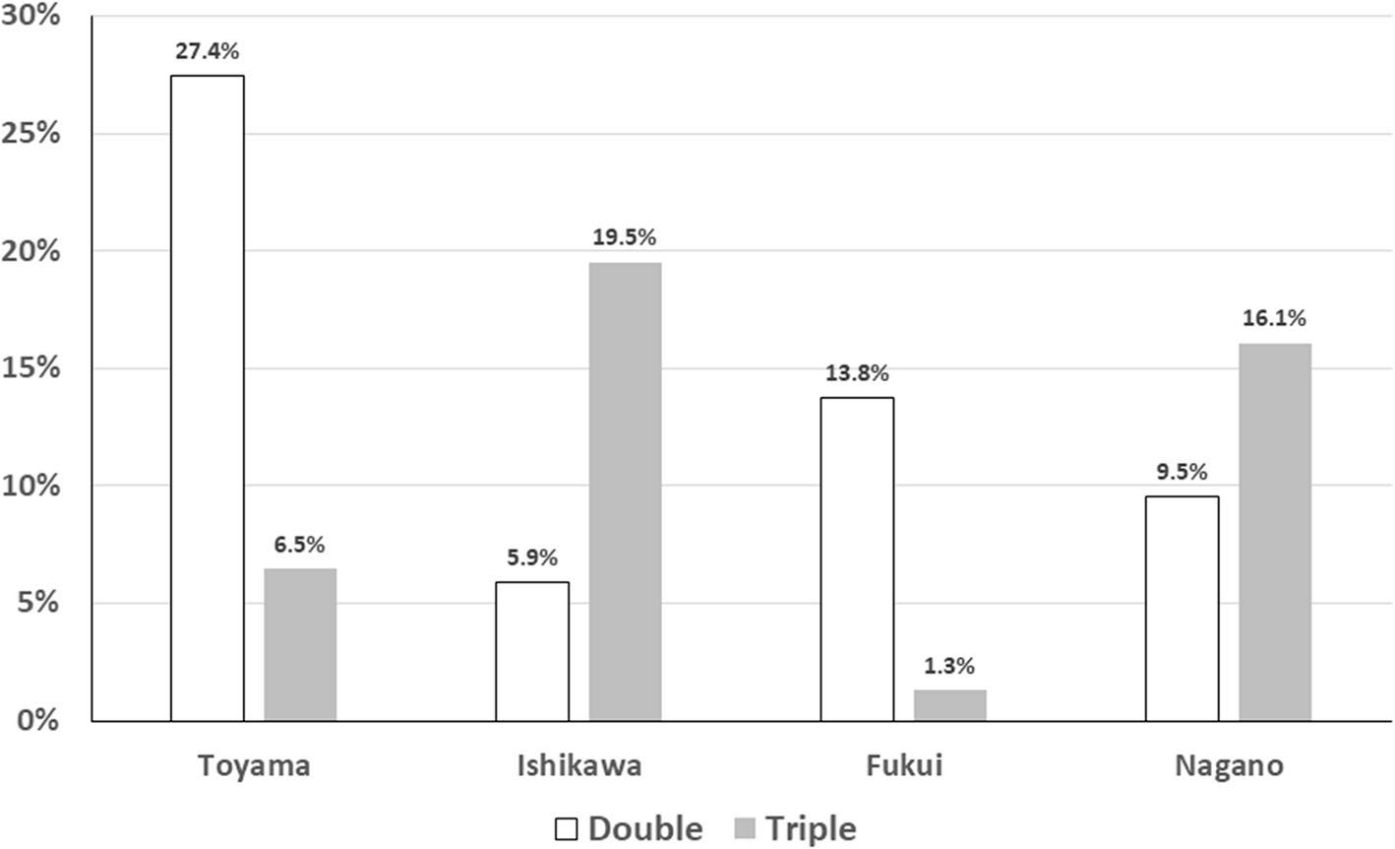
Comparison of frequencies of double and triple antiemetic therapy between four prefectures

In the present study, we analyzed the definition of malignancy corresponding to behavioral codes 2 or 3 in the International Classification of Disease for Oncology, 3rd edition (ICD-O-3). We analyzed the patients in Class of Cases 20 and 30 coded as 20 (diagnosed and treated in the registering hospital) and 30 (diagnosed in another hospital and treated in the registering hospital), respectively. UICC stages based on TNM (7^th^ edition) were used for stage classification. All targeted lung cancers (small cell lung cancer and non-small cell lung cancer) newly encountered at hospitals from January 1, 2016, to December 31, 2017, were analyzed. In addition, patients who were initially treated with carboplatin-based chemotherapy were enrolled. Patients receiving chemoradiotherapy were excluded from the present study. The interval of corresponding DPC data to HBCR in the Hokushin Ganpro Database was selected from October 1, 2015, to July 31, 2017.

Among agents combined with carboplatin, nab-paclitaxel was included as paclitaxel. Treatment regimens were classified as triple antiemetic therapy with 5-hydroxytryptamine-3 receptor antagonist, neurokinin-1 receptor antagonist, and dexamethasone and double antiemetic therapy consisting of 5-hydroxytryptamine-3 receptor antagonist and dexamethasone. This study was performed in line with the principles of the Declaration of Helsinki Approval and approved by the Institutional Review Board of Shinshu University School of Medicine (No.5054) and institutional review board approval was obtained from each participating facility for creating the database. The need for informed consent was waived by the Institutional Review Boards of Shinshu University School of Medicine (No.5054) and Kanazawa University (No.2750–4) due to the retrospective nature of the study and handling anonymized data. The dataset was used with permission from the Data Utilization Committee of Hokushin Ganpro Database Project. Chi-square test was used to compare the data.

## Results

### Patients

**Fig. 2:**
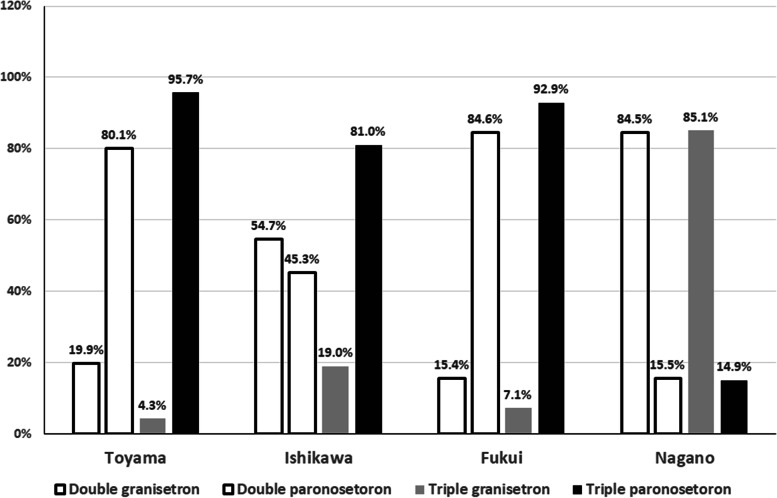
Comparisons of frequencies of first- (granisetron) and second-generation (palonosetron) 5-hydroxytryptamine-3 receptor antagonists in the double and triple antiemetic regimens between four prefectures

A total of 1,082 lung cancer patients treated with carboplatin, consisting of 861 (79.6%) men and 221 (20.4%) women with a median age of 69.4 years (range, 33 – 89 years), were included in the present study. Non-small cell non-squamous lung cancer was the most common cancer type (539 cases, 49.8%), followed by squamous cell carcinoma (288 cases, 26.6%) and small cell lung cancer (255 cases, 23.6%) (Table 1). There were no significant differences in male/female ratio or mean age among the four prefectures in the Hokushin area (Table 2). The cycles of carboplatin-based chemotherapy are also shown in Table 1. Four, five, and six cycles of treatment were applied in 37.2%, 5.9%, and 10.5% of cases, respectively. However, 157 (14.5%) and 128 (11.8%) cases received only one and two cycles of carboplatin-based chemotherapy, respectively, while 25 cases were treated with more than 10 cycles. The double and triple antiemetic regimens were prescribed for 613 (56.7%) and 469 (43.3%) patients, respectively. All patients received antiemetic therapy as recommended by the 2015 Japan Society of Clinical Oncology Clinical Practice Guidelines for Antiemesis, and therefore the rate of use of standard antiemetic therapy was 100% in Hokushin region. The rates of double and triple antiemetic therapy in the four prefectures are shown in Fig. 1. The rates of double antiemetic therapy were higher in Toyama and Fukui prefectures, while the rates of triple antiemetic therapy were higher in Ishikawa and Nagano prefectures. We analyzed the frequencies of first- and second-generation 5-hydroxytryptamine-3 receptor antagonists (granisetron etc. and palonosetron, respectively) in the double and triple antiemetic regimens. Palonosetron was used in 66.7% of cases receiving double antiemetics and in 59.1% of cases receiving triple antiemetics. The rates in the four prefectures are shown in Fig. 2. Interestingly, the rates of palonosetron use in double and triple antiemetic regimens were higher in Toyama and Fukui than in Ishikawa and Nagano. Nagano prefecture showed the lowest rates of palonosetron prescription in both double and triple antiemetic regimens.

**Table 1:**
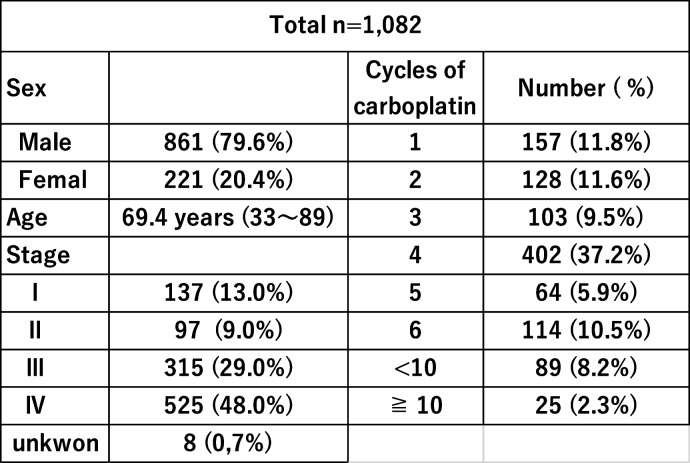
Patient characteristics and numbers of carboplatin-based chemotherapy cycles

**Table 2:**
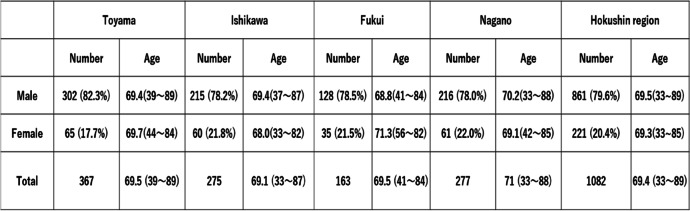
Numbers and mean ages of lung cancer patients in the four prefectures in Hokushin region

The rates of agents combined with carboplatin are summarized and compared among the four prefectures in Table 3. The main agents combined with carboplatin were paclitaxel, pemetrexed, and etoposide, accounting for approximately 85% of cases in both treated double and triple antiemetic therapy. Differences were observed among the four prefectures in rates of application of paclitaxel, pemetrexed, and etoposide between double and triple antiemetic regimens. Ishikawa and Nagano prefectures had higher rates of carboplatin combined with paclitaxel, pemetrexed, and etoposide in triple antiemetic therapy than in Toyama and Fukui prefecture, which showed the reverse result in double antiemetic therapy.

**Table 3:**
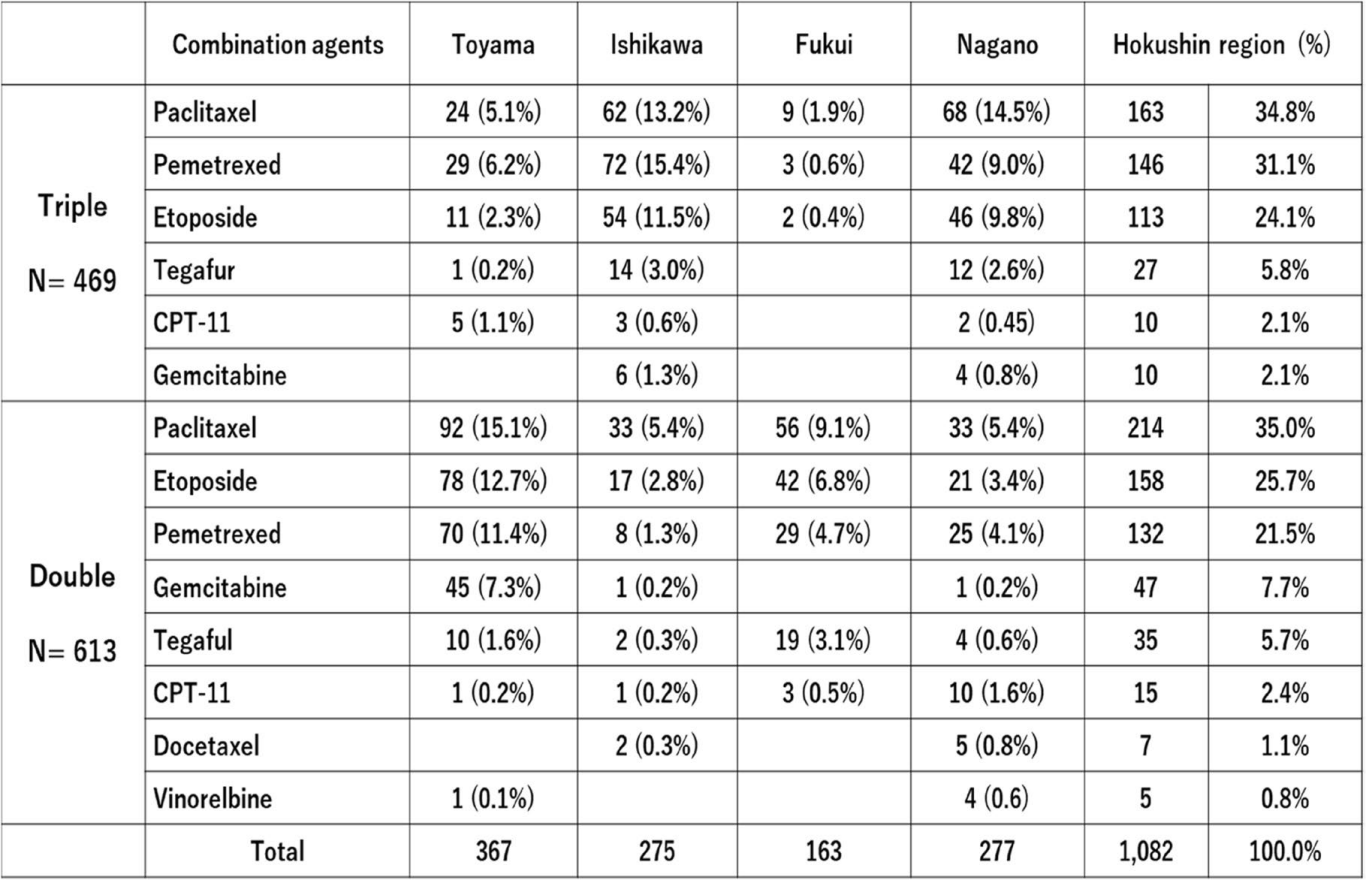
Chemotherapy regimens according to double and triple antiemetic therapy

Next, we examined the patterns of serial antiemesis after the second cycle of carboplatin-based chemotherapy (Table 4). The same antiemetics were prescribed in most patients (93%). However, four and 35 patients changed from double to triple antiemetic prophylaxis (3.6%), while five and 36 patients changed from triple to double antiemetic prophylaxis in the second and third cycles, respectively (3.8%). However, six of these patients returned to triple antiemesis in subsequent cycles. In addition, we examined the antiemesis in incomplete chemotherapy group (less 3 cycles of carboplatin administration). The rates of prescription of double and triple antiemetic prophylaxis were not significant different between the discontinuation group (less 3 cycles of carboplatin) and continuation group (greater 4 cycles of carboplatin) (52.4% vs. 59.0%, respectively, chi-square test). The number and rate in discontinuation group according to double and triple antiemetic regimens was shown in Supplemental Table 2 and the rate in four prefectures was shown in Supplement Table 3. There were no significant differences in the frequencies between double and three antiemetic regimens (Supplemental Table 2). Triple antiemetic regimen in Ishikawa and Nagano prefectures had higher rates in both less 3 cycles of carboplatin and greater 4 cycles than those in Toyama and Fukui prefectures, but total rate of double and triple antiemetic regimens was equal in both groups (Supplemental Table 3).

**Table 4:**
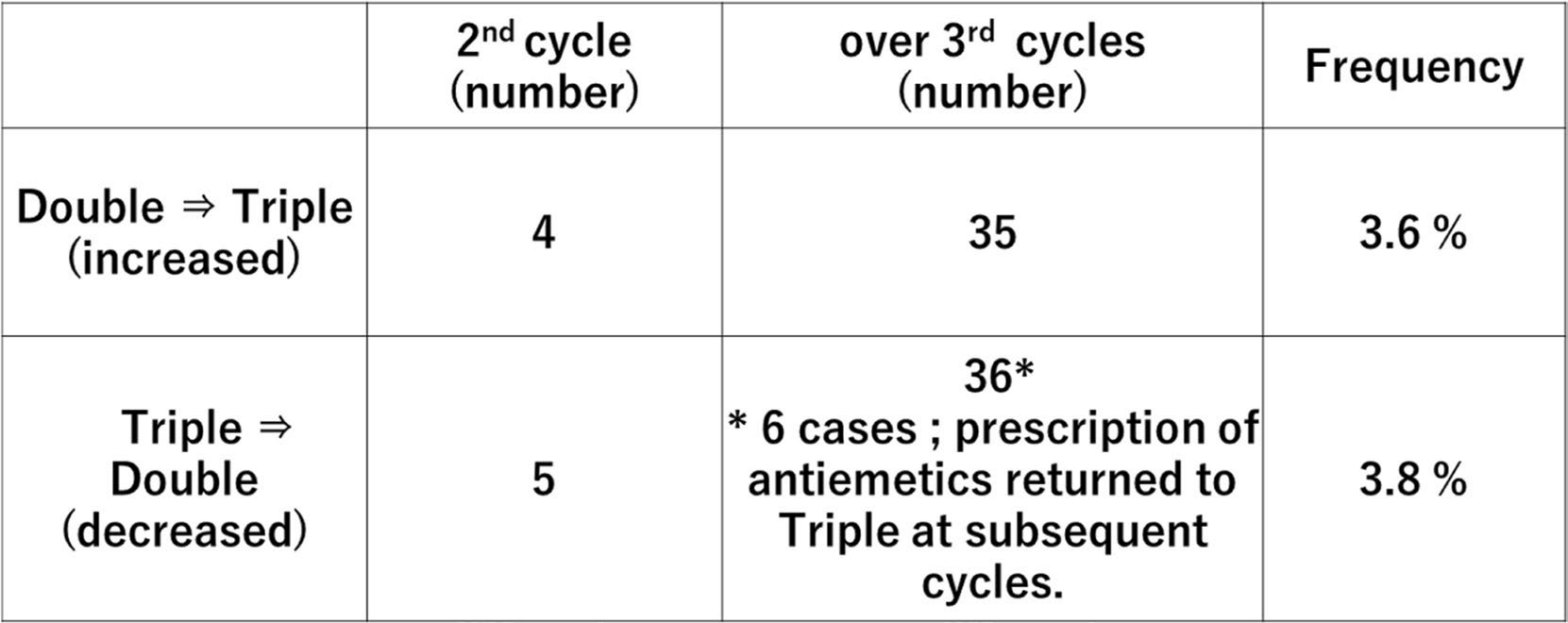
Changes in prescription of antiemetics

## Discussion

Here, we examined the antiemetic status for CINV among 1,082 newly diagnosed and treated lung cancer patients receiving carboplatin-based chemotherapy between 2016 and 2017 in the Hokushin region based on the Hokushin Ganpro dataset 2 consisting of HBCRs and DPCs. We found that prophylactic antiemetics were prescribed in all patients in accordance with the guidelines. The rate of double antiemetic therapy was slightly higher than triple antiemetic therapy, but the distribution and use of palonosetron were quite different between the four prefectures.

There have been several real-world cohort studies regarding the adherence to international clinical guidelines for CINV. Multiple prospective observational studies in the USA showed that the prevalence of guideline-consistent CINV prophylaxis was 73.1% in MEC regimens including carboplatin [15]. Aapro et al. [11] summarized the data in Global Oncology Monitor database (Ipsos Healthcare, London, UK), which collected patients’ medical charts from 610 representative physicians in five counties (France, Germany, Italy, Spain, and the UK). They showed that only 15% of all patients treated with HEC and carboplatin-based chemotherapy received guideline-recommended triple antiemetic prophylaxis. On the other hand, multicenter, prospective, observational studies in Japan showed that approximately 95% of patients treated with MEC (including carboplatin) received antiemetic therapy in compliance with the guidelines [8]. Based on these reports, double and triple antiemetics were prescribed in 67% and 28% of cases receiving MEC, respectively [8]. Subsequently, Okuyama et al. [10] summarized combined health service utilization and HBCR data and reported that 59.1% and 24.0% of patients treated with intravenous MEC received double and triple antiemetic prophylaxis, respectively, in Japan. Our results were focused on carboplatin-based chemotherapy and lung cancer. However, we found that 100% of patients were treated with prophylactic antiemetics in accordance with the guidelines. We believed that, based on the guideline, carboplatin regimen including antiemesis prescription was registered in advance in almost hospitals in Hokushin region. In addition, triple antiemetic therapy was prescribed in 46% of enrolled subjects in Hokushin region, which was a higher rate than in previous Japanese studies [8–10]. Recently, Iihara et al. [9] summarized the CINV pattern and status in patients treated with carboplatin-based chemotherapy and performed a comparison between double and triple antiemetic prophylaxis groups (69.4% and 30.6%, respectively). They suggested that triple antiemetic therapy was appropriate for antiemetic prophylaxis in patients with carboplatin-induced CINV because double antiemetic therapy was a risk factor for incomplete response of CINV. However, they also reported that there was no significant difference in control of CINV between double and triple antiemetic regimens in lung cancer patients [9]. Several studies showed that adding neurokinin-1 receptor antagonist improved carboplatin-induced CINV [6, 16], but the results were not consistent with other studies [17–19], including in lung cancer [9, 18, 19]. Therefore, although we found a relatively high rate of triple antiemetic regimen use in Hokushin region compared with other studies [8–10], further studies are needed regarding the effects of adding neurokinin-1 receptor antagonist to each carboplatin-based regime and different types of cancer.

Palonosetron is preferred to first-generation 5-hydroxytryptamine-3 receptor antagonists, such as granisetron or ondansetron, for MEC or HEC, and has proven useful for preventing both acute and delayed CINV [20]. Palonosetron was used dominantly in both double and triple antiemetic regimens in the present study. In addition, although double antiemetic therapy was prescribed frequently in Toyama and Fukui prefectures, palonosetron was mainly selected as the 5-hydroxytryptamine-3 receptor antagonist in these prefectures, which was reasonable for better prophylaxis for CINV. In contrast, palonosetron was used less often in Nagano prefecture. Although the reasons were not clear, but these real-world data were informative for reviewing cancer management in this region.

It is noteworthy that 39 patients (3.6%) changed from double to triple antiemetic therapy after the second cycle of carboplatin treatment in the present study. In contrast, 41 patients changed from triple to double antiemetic therapy after the second cycle of carboplatin treatment, but six of these patients returned to the triple regimen at subsequent cycles. We were unable to evaluate the severity of CINV in each patient, but these data suggested that possibly maximal CINV prevention was required even in cases of carboplatin-based chemotherapy. It was reported that the carboplatin + pemetrexed regimen, which is commonly used in lung cancer, had a higher risk of causing delayed nausea than the carboplatin + paclitaxel regimen that is widely used in various cancers [21]. Therefore, the optimal antiemetic therapy for lung cancer patients should be determined carefully, even for carboplatin-based chemotherapy. The optimal preventive antiemetic regimen for lung cancer patients receiving carboplatin remains unclear. However, optimal antiemetic prophylaxis should be considered according to the cancer type and regimens in patients treated with carboplatin-based chemotherapy.

The present study had some limitations. First, this was a retrospective review with a relatively small population and we were unable to evaluate the baseline characteristics, including patient performance status, comorbidities, etc. In addition, this study did not assess the status and/or frequency of CINV in each patient. Thus, it was unclear whether the discontinuation of chemotherapy and changed from double to triple antiemetic therapy was due to poor control of CINV. At least, we need further study to analyze CINV status in detail in cases that were changed from double to triple antiemetic therapy. The Hokushin Ganpro Database does not necessarily contain all HBCR data for the Hokushin region. Therefore, care should be taken in comparing double and triple antiemetic prophylaxis between prefectures. Furthermore, the present study was performed using data of 2016–2017, comparison of historical and/or serial analyses could be useful information for physicians. Nevertheless, our survey data corresponding to each registered case in the Hokushin Ganpro dataset provided the details of real-world practice and could be helpful in understanding the real-world clinical situation regarding treatment and management of lung cancer in the Hokushin region.

In conclusion, we described clinical practice for prevention of CINV related to carboplatin chemotherapy in patients with lung cancer in the Hokushin region, Japan. This area showed an extremely high adherence to antiemetic guidelines. However, antiemetic patterns differed between the four prefectures in Hokushin region, that could contribute to raise awareness for physicians in the management and prevention for CINV during carboplatin chemotherapy.

## Supplementary Information

**Additional file 1.**

**Additional file 2.**

**Additional file 3.**

**Additional file 4.**

## Data Availability

All data generated or analyzed during this study are included in this published article and its supplementary information files.

## Abbreviations

CINV: Chemotherapy-induced nausea and vomiting
HEC: Highly emetogenic chemotherapy
MEC: Moderately emetogenic chemotherapy
HBCR: Hospital-based cancer registry
DPC: Diagnosis procedure combination
