# Supplementary material for: Multi-institutional survey of antiemetic therapy in lung cancer patients treated with carboplatin in Hokushin region
Source: BMC Pulm Med. 2023 Jun 26;23:228. doi: 10.1186/s12890-023-02524-2 (PMC10294304; doi:10.1186/s12890-023-02524-2)
Supplement: Supplementary file 1 — Additional file 1. [file 12890_2023_2524_MOESM1_ESM.docx]

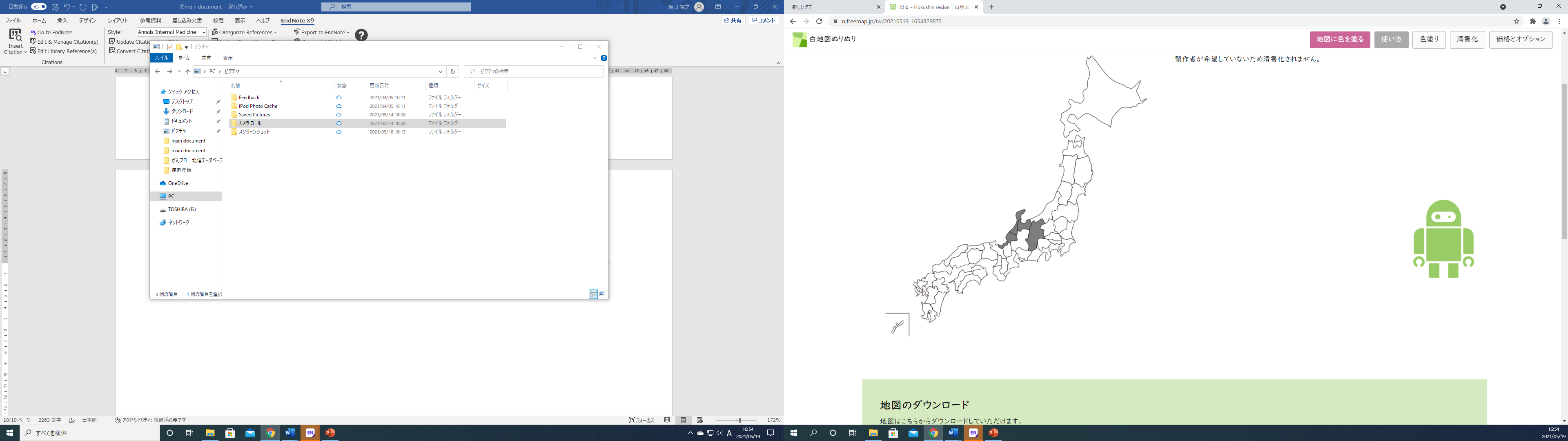


Fukui

Ishikawa

Toyama

Nagano

**Hokushin region**


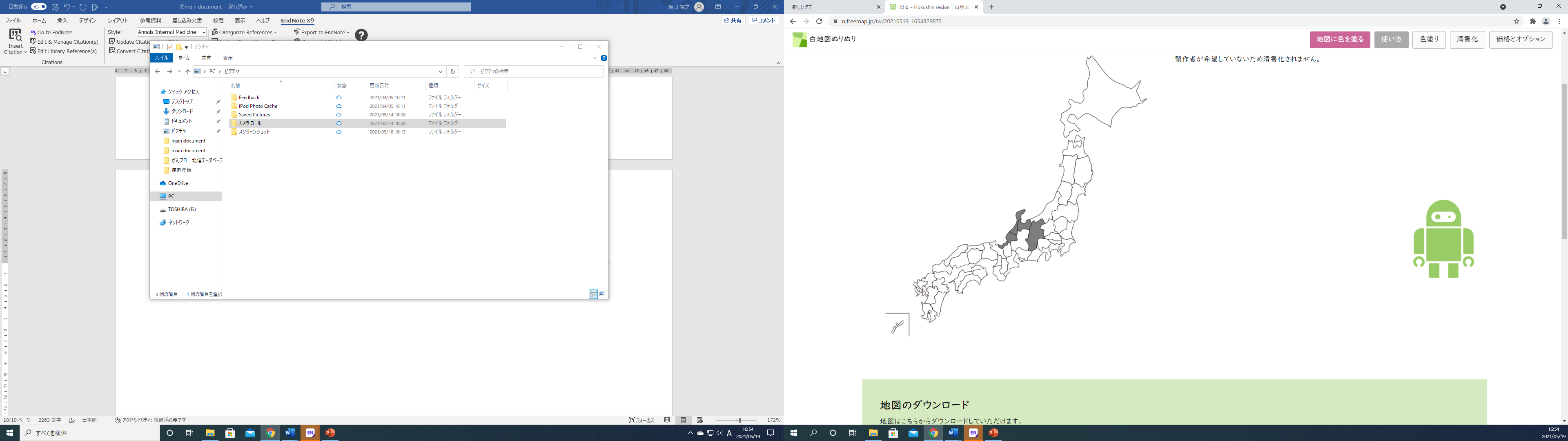


Fukui

Ishikawa

Toyama

**Hokushin region**

**Supplementary Figure 1.** The Hokushin region is composed of the Fukui, Ishikawa, Toyama, and Nagano prefectures, which have commonalities, such as the population’s age distribution and snowy climates during the winter.
