# Supplementary material for: Multi-institutional survey of antiemetic therapy in lung cancer patients treated with carboplatin in Hokushin region
Source: BMC Pulm Med. 2023 Jun 26;23:228. doi: 10.1186/s12890-023-02524-2 (PMC10294304; doi:10.1186/s12890-023-02524-2)
Supplement: Supplementary file 2 — Additional file 2. [file 12890_2023_2524_MOESM2_ESM.docx]

| Fukui prefecture | Fukui University Hospital | Ishikawa prefecture | Kanazawa University Hospital |
| --- | --- | --- | --- |
|  | Fukui Prefectural Hospital |  | Kanazawa Medical University Hospital |
|  | National Hospital Organization Tsuruga Medical Center |  | Ishikawa Prefectural Central Hospital |
| Toyama precfecture | Toyama University Hospital |  | National Hospital Organization Kanazawa Medical Center |
|  | Toyama City Hospital | Nagano precfcture | Shinshu University Hospital |
|  | Kurobe City Hospital |  | Nagano Municipal Hospital |
|  | Toyama Rosai Hospital |  | Nagano Red Cross Hospital |
|  | JA Toyama Kouseiren Takaoka Hospital |  | Suwa Red Cross Hospital |
|  | Takaoka City Hospital |  | Nagano Children’s Hospital |
|  | Tonami General Hospital, |  | Aizawa Hospital |
|  | Toyama Prefectural Central Hospital |  |  |

**Supplementary Table 1.**

Lists of participating hospitals in the present study.
