# Supplementary material for: Multi-institutional survey of antiemetic therapy in lung cancer patients treated with carboplatin in Hokushin region
Source: BMC Pulm Med. 2023 Jun 26;23:228. doi: 10.1186/s12890-023-02524-2 (PMC10294304; doi:10.1186/s12890-023-02524-2)
Supplement: Supplementary file 3 — Additional file 3. [file 12890_2023_2524_MOESM3_ESM.docx]

Supplement Table 2

The number and frequency in discontinuation group (less than three cycles of carboplatin ) according to double and triple antiemetic regimens.

| **Treatment cycle in discontinued cases** | **Antiemetic regimens** | **Number** | **%** |
| --- | --- | --- | --- |
| **One** | **Double** | **82** | **52.6** |
|  | **Triple** | **74** | **47.4** |
| **Two** | **Double** | **69** | **53.1** |
|  | **Triple** | **61** | **46.9** |
| **Three** | **Double** | **53** | **51.0** |
|  | **Triple** | **50** | **49.0** |
