# Supplementary material for: Multi-institutional survey of antiemetic therapy in lung cancer patients treated with carboplatin in Hokushin region
Source: BMC Pulm Med. 2023 Jun 26;23:228. doi: 10.1186/s12890-023-02524-2 (PMC10294304; doi:10.1186/s12890-023-02524-2)
Supplement: Supplementary file 4 — Additional file 4. [file 12890_2023_2524_MOESM4_ESM.docx]

Supplement Table 3

The rate according to double and triple antiemetic regimens in four prefectures.

| **Treatment Cycles** | **Antiemetic regimens** | **Toyama** | **Ishikawa** | **Fukui** | **Nagano** | **Hokushin** |
| --- | --- | --- | --- | --- | --- | --- |
| **Less than three cycles** | **Double** | **26.9%** | **7.6%** | **32.5%** | **11.2%** | **18.9%** |
|  | **Triple** | **6.5%** | **29.1%** | **3.7%** | **27.0%** | **17.1%** |
|  | **Total** | **33.5%** | **36.7%** | **36.2%** | **38.3%** | **36/0%** |
| **Greater 4 cycles** | **Double** | **54.0%** | **15.6%** | **58.9%** | **26.1%** | **37.8%** |
|  | **Triple** | **12.5%** | **47.6%** | **4.9%** | **35.7%** | **26.2%** |
|  | **Total** | **66.5%** | **63.3%** | **63.8%** | **61.7%** | **64.0%** |
